# Covid-19 and Families With Parental Mental Illness: Crisis and Opportunity

**DOI:** 10.3389/fpsyt.2021.567447

**Published:** 2021-07-27

**Authors:** Mairead Furlong, Sinead McGilloway, Christine Mulligan, Mary G. Killion, Sharon McGarr, Anne Grant, Gavin Davidson, Mary Donaghy

**Affiliations:** ^1^Centre for Mental Health and Community Research, Maynooth University, Kildare, Ireland; ^2^Health Service Executive Galway, Roscommon Adult Mental Health Services, Galway, Ireland; ^3^School of Nursing, Queen's University, Belfast, Ireland; ^4^Praxis Chair of Social Care, School of Social Sciences, Education and Social Work, Queen's University Belfast, Belfast, Ireland; ^5^Mental Health & Learning Disability Lead & Think Family NI Lead, HSC Board, Belfast, Ireland

**Keywords:** children, COVID-19, family, mental health, mental illness, parents, pandemic, mental disorder

## Abstract

The COVID-19 emergency has affected us all, but not equally. Families where parents have mental illness (PMI) are potentially at increased risk, but little is known about how they or their support services managed under lockdown/restrictions. We harnessed our existing partnerships with adult and child mental health services in the Republic of Ireland (RoI) and Northern Ireland (NI) to investigate the qualitative experiences of service users and families in coping during the first COVID-19 lockdown (March–May 2020), and how services were supporting them. Semi-structured phone/online interviews were conducted with 22 clinicians/managers (12 from RoI; 10 from NI) who provided information from their caseloads (~155 families with PMI). Sixteen family members (10 from RoI, 6 from NI) were also interviewed. Data were analysed using standard thematic analysis. Sixty percent of families reported improved mental health, primarily due to respite from daily stresses and the “normalisation” of mental distress in the general population. Approximately 30%, typically with more severe/enduring mental illness, reported additional challenges, and mental distress including: unmanageable child behaviours; fear of relapse/hospitalisation; financial difficulties; absence of child care; and a lack of routines. Service provision varied considerably across regions. The experiences within this case study highlight unique opportunities to address the multiple stresses of pre-emergency daily living. We also highlight how mental health services and governments might become more “pandemic ready” to more effectively support vulnerable families, including addressing service overload issues, optimising the use of digital technologies, and providing in-person contact and social supports where required.

## Introduction

On the 11th March 2020, the World Health Organisation declared the novel coronavirus (COVID-19) a pandemic, with immediate, global impact on our daily lives (1). As infections escalated during the first wave of the virus, the impact of lockdown/public health restrictions on mental health in the general population was increasingly recognised, as well as the need to support parents and children (2, 3). A UK population-based survey conducted in April 2020 highlighted greater concerns about the psychosocial effects of the emergency (e.g., stress, anxiety) than the risk of infection (4). Other similar surveys in the UK, US, and China found that 62% (5), 56% (6), and 54% (7) of respondents reported moderate to severe levels of pandemic-related emotional distress during this time, including anxiety, stress, increased substance misuse, sleep difficulties and emotional regulation difficulties. Key triggers included: disrupted social networks/isolation; pressurised home environments (e.g., from overcrowding, home working, and lack of childcare); income loss/insecurity; and fear of infection.

Despite international calls to investigate the impact of COVID-19 on vulnerable groups (2), far less research has examined the experiences of those with pre-existing mental illness, socially disadvantaged families, or the services that support them. During the first, and subsequent lockdowns, there was increased alcohol consumption in 27% of family households (8, 9), and EU states (including Ireland and the UK) experienced a 60% increase in domestic violence-related calls (10). Furthermore, child protection referrals decreased by 35–50% during the first lockdown (March to June 2020), suggesting that cases were not being identified due to the closure/restrictions in usual referral sources (e.g., schools, General Practitioners and allied health professionals) (11, 12).

A UK study conducted during the first lockdown showed that levels of anxiety, depression, loneliness, and thoughts of self-harm were higher among people with pre-existing mental illness than in the general population (13). However, it is not known how many survey respondents were parents with dependent children. Internationally, a “hidden population” of one in four children have a parent with mental illness (PMI) and Northern Ireland (NI) currently has the highest levels of maternal mental illness within the UK (14), experienced by 30% of children aged 0–16 years. Despite substantial evidence indicating the intergenerational transmission of mental illness (15), the complex needs of these children and families typically go un-recognised and untreated (16).

Currently, most mental health services do not recognise the parenting status of service users due to individualised approaches to assessment and treatment, segregation of adult and child, and adolescent mental health services (AMHS/CAMHS) and professional competencies around child welfare concerns (17). Therefore, family-focused mental health initiatives, such as COPMI^1^ in Australia and the UK Think Family Initiative (including NI) (18), were established to promote public/service awareness of the need to identify and support these “hidden” families (19, 20). However, other countries, including the RoI, lack basic policy guidance on how to identify and support families with PMI (21, 22).

In 2017, the Health Services Executive in the RoI funded the “PRIMERA”^2^ research programme (2017–2021) to help implement and evaluate family-focused practise for families with PMI (randomised controlled trial (RCT) involving 92 families [152 parents, 249 children]) in 15 sites across the RoI and involving adult, child, and primary care mental health services (23, 24). Eighty percent of the PRIMERA parent sample attend AMHS for various mental health disorders; 20% receive antidepressant medication or primary care psychological support (24). This research provided the impetus for this paper.

Given the sudden and dramatic changes related to COVID-19, we were keen to ascertain how services and families with PMI in two different jurisdictions (the RoI and NI) were responding to stringent emergency-related restrictions. The objectives of this article were to explore: (1) the experiences of service users and families with PMI during the first COVID-19 lockdown; and (2) the views/experiences of mental health service providers who were supporting these families.

## Method

### Participants

We utilised (and sought permission from) our existing adult and child mental health service partners from the PRIMERA research and the Think Family initiative in NI (TFNI) to investigate clinician experiences on how services were supporting families with PMI during the first COVID-19 lockdown (March to end of May 2020), and to explore with a sample of such families how they were coping during this time.

In the RoI, service-provider participants included 12 clinicians/managers, linked to 8 PRIMERA sites, and working across AMHS, CAMHS, and primary care outpatient mental health services. They provided information on how families with PMI on their caseloads (~70 families) were coping during the first lockdown and on their own experiences of supporting these families (45 of the families were also participants in the PRIMERA RCT). Clinicians were predominantly from the disciplines of social work and social care (*n* = 7), with reports also from three psychologists, one clinical nurse, and one family therapist. Family participants included 10 parents (6 PMI, 4 partners of PMI) and were purposively selected from the PRIMERA RCT sample (*n* = 92). All families (including the 70 reported upon by clinicians and the 10 who provided direct report) had a similar profile to our larger RCT sample in terms of age and gender (88% female, aged 26–50 years), mental health disorder, site/location and (mainly socially deprived) socioeconomic status. Within the PRIMERA RCT sample, anxiety and/or depression are the most common mental health challenges (64%), followed by bipolar disorder (18%), emotional regulation disorder (10%), psychosis (6%), and Post Traumatic Stress Disorder (PTSD; 2%). At baseline, on average, families reported clinically significant emotional, interpersonal and family distress.

In NI, 10 Assistant Directors/psychiatrists, representing the views of acute adult mental health services across NI, provided feedback to the TFNI Coordinator (MD) on clinician and service user experiences (*n* = 85) during the first lockdown (Donaghy, personal communication 27.05.2020). Data on the prevalence and severity of mental illness presentations are not yet routinely collected in acute adult mental health services in NI (25). Assistant Directors/psychiatrists estimated that the most common diagnoses are anxiety and depression, substance misuse disorders, bipolar disorder, PTSD and psychoses, which is consistent with research on prevalence of psychopathology in NI (26). We do not have accurate age or gender data for this sample but it estimated that services users with PMI are mainly female and aged 25–55 years old. Family participants in NI (*n* = 6) were service users from one heath care Trust in NI, female (*n* = 5), aged 30–50 years old. Their diagnoses included anxiety and depression (*n* = 4), bipolar disorder (*n* = 1) and emotional regulation disorder (*n* = 1). Their experiences were seen as typical of service-user experiences in NI more generally (Donaghy, personal communication 27.05.2020).

### Data Collection and Analysis

**Figure 1:**
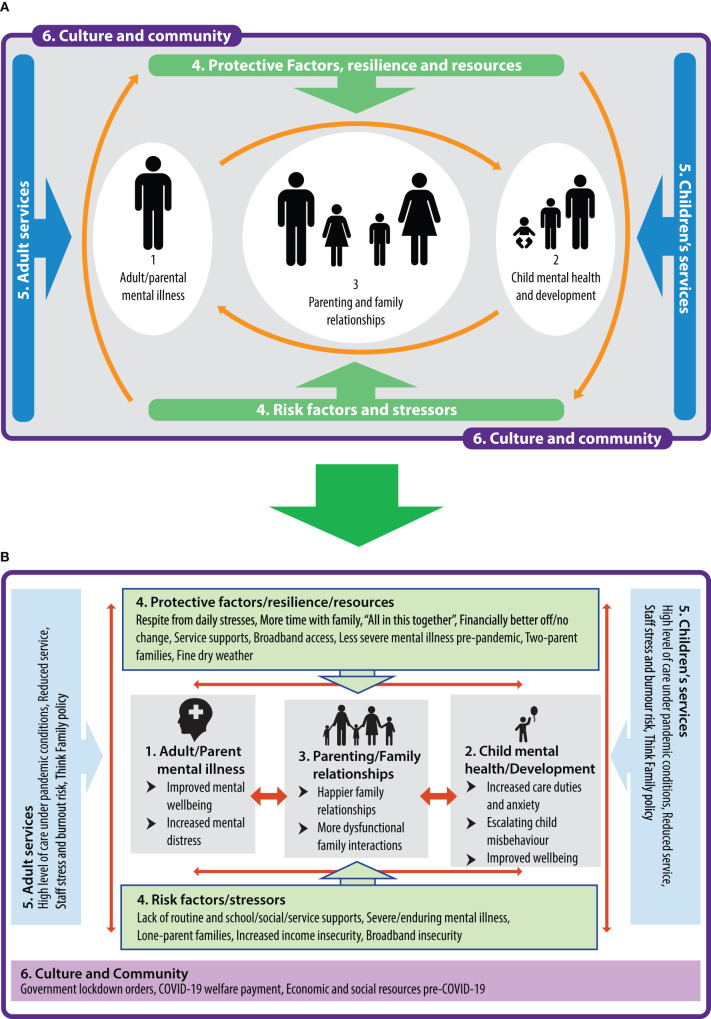
**(A)** The family model (16). **(B)** Qualitative themes contextualised within framework of the family model.

Semi-structured one-to-one phone interviews—lasting 15–30 min—were conducted with families (*n* = 16) in the RoI and NI to explore their perceptions of how the COVID-19 emergency had impacted parent and family mental health and well-being. Likewise, service providers (*n* = 22) in the RoI and NI provided feedback (via phone, email, and online platforms) on how ~155 families with PMI on their caseloads, were coping during the lockdown, as well as on their own experience of supporting these families. Detailed notes were taken from interviewees and due to time constraints, were subjected to selected verbatim transcription. Data were analysed using Braun and Clarke's thematic analysis to identify key themes across both jurisdictions, involving the process of familiarisation, coding, and generating, reviewing and naming themes (27). This process was framed and contextualised using The Family Model (28) (Figure 1), which highlights the interconnections among parent, child and family mental health, service responses, cultural influences, and protective and stressor factors within a transgenerational approach to mental health recovery.

The study was approved by Maynooth University Social Research Ethics Committee.

## Results

Sample quotes for identified themes can be seen in the Supplementary Table 1.

### Improved Family Mental Health or No Change During Lockdown

Unexpectedly, both clinicians and families indicated that most families (~60%) reported substantially improved mental health and family relationships during this first lockdown, despite having a mental health diagnosis.

#### Respite From Stresses of Daily Life and Time to Connect With Family

A key factor was the respite from the stresses of daily life. The slower pace of life, with less pressure to be up and out early in the morning for work, school, and childcare, as well as minimal service appointments and extracurricular activities, meant that about 60% of parents and children felt more rested and relaxed during the lockdown, and according to clinician and family reports, experienced less stress, anxiety and depression. Several parents (*n* = 7) indicated that they now had time to “*just be themselves*.” Other parents (*n* = 5) recounted benefitting from more emotional and childcare support from partners due to home-based remote working. Ten parents also reported that they had more time to connect with their children (e.g., through cooking, walking, and gardening) and that this, combined with less school and exam pressures, had led, for the first time, to more open and honest communication from their children:

“*He has opened up so much about how he feels about us separating and that is definitely due to the time we're spending together now.”*

“*I didn't realise how much stress commuting was doing to us until it stopped.”*

Both clinicians (*n* = 13) and parents (*n* = 6) indicated that family conflicts were more easily resolved, and had led to subsequent feelings of parental empowerment. A number of clinicians noted (*n* = 9) that positive experiences of lockdown were more common in two-parent families and with less severe PMI, although several service users with psychosis and bipolar disorder also reported improved mental health.

“*Taking away the daily pressures of school, exams and work means that families are able to connect better with each other. There are less triggers for conflict, and when there is conflict, they seem more able to reach a positive resolution. For some parents they have become more confident in themselves, more empowered. This seems to be good for everyone's mental health.”* (Clinician, RoI).

#### A Chance to Feel Normal

Confinement in lockdown and fears of infection meant that anxiety and other mental health concerns became normalised and, consequently, many parents (*n* = 10) reported that they felt less alone and more “*normal*” in expressing anxiety “*as the whole world is in crisis*.” Several clinicians (*n* = 8) indicated that many parents were now feeling less stigmatised and were more openly discussing their mental health issues. This, in turn, was seen as beneficial for their own coping and parent-child relationships. Several parents (*n* = 7) also reported that they enjoyed the respite from attending multiple service appointments and felt less monitored and judged as a result.

“*I'm surprised by how well I'm doing and even better than some people I know who don't attend [mental health] services. I can just be myself, take time to look after myself. I feel less judged. The phone calls with my addiction counsellor are helping.”* (Parent).

“*The recognition that the crisis is having a psychological impact on the general population has given some parents permission to discuss their own mental health needs more openly, with Covid as the protagonist.”* (Clinician, RoI).

#### No Change

A small number of parents (*n* = 2) indicated that lockdown had not affected their mental health. Current family routines were largely similar to pre-emergency conditions in terms of one parent going to work whilst the service-user parent stayed at home with the children. However, the experience of lockdown may change over time. For instance, three parents reported that the initial stages were more difficult, but were coping better now. Conversely, others (*n* = 3) were finding the restrictions increasingly challenging over time.

### Families Struggling During Lockdown

#### Increased Family Distress Due to Worsening Parental Mental Illness

Clinician and family reports indicated that ~30% of families were experiencing substantial additional challenges due to lockdown restrictions, notably in cases involving more severe/enduring PMI. Mental distress was exacerbated in instances where a parent was at risk of relapse and hospitalisation. For example, a clinician reported that within one lone-parent family, extended family members would usually step in to provide childcare, but instead, the children were undertaking inappropriate levels of care/work to help prevent their mother's relapse. The mother expressed concerns about where her children could stay if she were hospitalised as well as the potentially increased risk of infection from hospital admission.

“*Families with one parent are finding this period very difficult especially if they feel they're relapsing. They have the added worry of who is going to look after the childre*n.” (Clinician, NI).

More than half of clinicians (*n* = 12) reported that lockdown increased anxiety and irritability for some parents, which they projected onto their children or partners. Correspondingly, two partners of service-user parents reported struggling with the increased tension and hostility in the home, lack of social outlets and reduced mental health service supports. A number of clinicians (*n* = 6) and families (*n* = 4) indicated increased alcohol use as a coping measure. Five clinicians observed that parents who have emotional regulation difficulties were particularly struggling. These parents missed their usual routines and having time away from their children, thereby leading to more strained relationships with their children and partners. In both the RoI and NI, clinicians (*n* = 8) advised that there appeared to be a deterioration in client mental health as lockdown continued, with increased case complexity and a rise in referrals as time went on.

“*Where a parent has emotional dsyregulation, they are struggling with not being able to get respite from children and home life.”* (Clinician, RoI).

#### Escalating Child Misbehaviour

Four parents reported that child externalising misbehaviour (e.g., disobedience, conflict) had escalated during lockdown which, in turn, contributed to parental mental distress. The lack of routine and socialising activities for children was very challenging for some parents in managing misbehaviour. Two parents indicated that the child welfare and protection service in the RoI (Tusla) was involved. One parent compared the home atmosphere to “*a volcano*” while another said: “*We're literally breaking apart*.” Similarly, five clinicians described volatile home situations where Tusla involvement was required.

“*I didn't think it was going to be this hard. With the schools closed, I can't manage the [five] children. We're literally breaking apart…Tusla are involved.”* (Parent).

#### Protecting Others From Increased Stress

A number of parents (*n* = 3) and clinicians (*n* = 9) reported that the lack of childcare support, and expectations of working from home while home schooling, had exacerbated parental stress, but that parents were working with services to mitigate negative effects on their families. Five parents indicated they were experiencing financial difficulties and feared job losses. While parents were trying to protect their children from their financial (and other) worries, one clinician noted that children were also trying to manage their own mental health concerns so as not to overburden their parents. This is commonly seen in families with PMI, where communication of anxieties may be perceived as compounding family burden (29).

“*We are finding it very hard financially with me not allowed to work and schools closed. The kids are not doing too bad but we [parents] are struggling.”* (Parent).

#### Coercive Controlling Behaviour

There were also some examples of abusive relationships from clinician and family reports. For example, one parent with pre-existing depression indicated that her partner regularly flouted the physical distancing rules and left her alone while pregnant to look after their 7-month-old baby while he went drinking. She was in tele-contact with a psychologist to help her manage the coercive controlling dynamics of the relationship.

### Service Responses and Staff Well-Being

There was considerable variation across regions in NI and the RoI in terms of mental health service capacity to respond to families. Clinicians in some adult and primary care mental health services in both NI and the RoI were partially redeployed to frontline COVID-19 duties (e.g., covid testing), and several (*n* = 6) reported high levels of stress and burnout risk due to a lack of childcare support, being redeployed and on call for frontline COVID-19 duties, and a belief that phone support alone was insufficient to meet the needs of many of their clients:

“*We are providing 'essential' services which is extremely important for public health safety due to COVID-19, but does feel inadequate from a mental health perspective.”* (Clinician, RoI).

Four clinicians in the RoI questioned the strategy of redeploying short-staffed mental health teams to frontline swabbing duties. In other areas—both in NI and the RoI—crisis child protection services were reduced, and were not intervening in volatile situations where children were at risk of entering care.

“*Foster placements are breaking down with no crisis intervention, and young people are being transferred to residential care. Social workers are overwhelmed.”* (Clinician, RoI).

Clinicians (*n* = 10) in both jurisdictions also reported more positive experiences. One CAMHS area in the RoI, covering four counties, reported that phone support was well-received by families. Two AMHS areas (straddling three counties) provided high levels of tele-psychiatry support to families due to fewer new referrals than usual. Phone support reportedly worked best in a context of pre-existing relationships with families, although most clinicians (*n* = 14) believed that, in the longer term, face-to-face support would be required and that risk assessments were more difficult to conduct remotely. Commendably, within the two AMHS areas noted above, clinicians continued to undertake home visits and outpatient clinics for more vulnerable patients during the first lockdown, using COVID-19 guidelines. In addition, clinicians in these areas liaised with community organisations to help with service users' food and medication needs. While AMHS and CAMHS in NI were reportedly utilising online technologies during the emergency (Department of Health, 2020), very few of the PRIMERA AMHS and CAMHS sites were using virtual supports by June 2020. However, considerable progress has been made since then: during subsequent lockdowns, online and video platforms for individual and group interventions were commonly provided as part of service delivery in most PRIMERA sites, along with in-person support when required.

“*Telephone support has been received well by most service users. We have been able to provide more intensive telephone support as the rate of new referrals to adult mental health services has more than halved since the Covid restrictions. We have also been able to arrange home visits and out-patient clinics where required, using Covid guidelines.”* (Clinician, RoI).

## Discussion

This paper provides an important snapshot in time of families with PMI during the first lockdown (March–May 2020) in two neighbouring jurisdictions. The observation that most families experienced improvements, or no change in their mental health, was unexpected, especially in view of the generally negative effect of COVID-19 on adult mental health in the general population (5).

For most (including some with severe/enduring mental illness), the lockdown provided a welcome respite from the stressors of “normal” daily life including school runs, service appointments, extracurricular activities and long commutes to work, all of which led to parents feeling more rested/relaxed, with considerable benefits for the entire family. Likewise, the results of a small study in one CAMHS area in the RoI, indicated that clients reported improved mental health during the first lockdown (30). Other research has also found that confinement benefitted those with schizophrenia and eating disorders (31–33), although others have reported adverse effects (34). Within the context of the current study, most of our feedback concerned families of lower socioeconomic status, and consequently, some furloughed workers (especially those in the RoI) were possibly benefitting from the COVID-19 unemployment payment; in addition, families more dependent on welfare benefits may be less affected by the immediate economic implications of the emergency (e.g., potential job loss). Further contextual factors, such as the unusually fine weather in Ireland and the UK during the first lockdown, also contributed to a “semi-holiday” experience for some families. Several families also reported feeling more “normal” due to a perception that mental distress was being experienced more widely in the population.

Disaster research suggests that crises may prompt responses of psychological resilience (35), and certainly a spirit of “battening down” to conquer the virus was evident among our family participants. However, it is less clear as to whether or not resilience efforts can be sustained indefinitely across a series of lockdowns. It is likely that many families in our study experienced a temporary “cocooning” effect from the “normal” hassles of daily life in the first lockdown and were not yet experiencing the probable longer-term negative economic and educational implications of the emergency. Life may become more stressful for families as countries emerge from lockdown and daily responsibilities return. Many commentators anticipate severe unemployment, especially with regard to low-income jobs less amenable to remote working (e.g., tourism, retail), and increased risk of mental illness and suicide, with services unable to meet expected demand (5, 36). More recent research supports this view, indicating a decline in referrals to mental health services during the first lockdown, but increased numbers and case complexity several months later (37, 38). In addition, the results of a survey of adult mental health service users in the RoI undertaken from May to August 2020, showed increased anxiety and isolation, and less engagement with services (39). Notably, the sample in the current study differed in that they were engaged with family-support services during lockdown due to their involvement in the PRIMERA research in the RoI and being part of the “Think Family” initiative in NI.

While the health, economic, and psychosocial impact of the COVID-19 emergency constitutes a major crisis, the positive experiences of many families during lockdown (all with pre-existing mental health challenges) nevertheless provide a unique opportunity to rethink how we can better structure our “normal” lives to minimise the stresses of early starts, long commutes, and work/family imbalances. Many sectors/governments (e.g., New Zealand) are considering the feasibility and benefits of remote digital working, 4-day weeks, and staggered commuting to reduce the risk of contracting and spreading COVID-19 (40, 41). Microsoft Japan have asked companies to join them in repeating their 2019 4-day-week experiment where they reported a 40% increase in productivity, with fewer sick days, happier, more motivated staff and a 23% reduction in electricity usage (42). Such measures could help mitigate COVID-19 infection, whilst also creating better work/family balance.

We were struck by the observation that service overload may contribute, at least in part, to family distress. Several families reported a sense of relief due to less surveillance (and implicit judgement) from services (although all were receiving some level of phone (and other) support by family-focused clinicians during the lockdown). Indeed, an ongoing NI case-file audit has found an average of 9.2 services (ranging from 1 to 23) working with these families (43). This suggests a need for services to avoid duplication and to consider more carefully the optimal number of appointments for families in a given week/month. However, while families enjoyed a temporary respite from attending services, a UK Department of Health (2019) report indicated that child health/development is likely to be significantly impaired without such supports in the longer term (44). In the RoI—and as confinement continued—service users reported increased anxiety and isolation due to less face-to-face engagement with mental health services (39).

Notwithstanding the mental health benefits of lockdown for many families in the current study, a substantial proportion of families, typically with more severe/enduring mental illness or emotional regulation difficulties, experienced additional challenges and mental distress due to confinement/isolation, lack of routines, anxiety, substance misuse, and child externalising issues. This is similar to reports highlighting an increased risk of additional mental distress and financial difficulties for the more socially disadvantaged, as well as increased alcohol misuse and domestic violence during lockdown (5, 10). Furthermore, we found substantial variation in the support provided to families across service areas, including management decisions on allowing safe in-person contact and redeploying clinicians to frontline COVID-19 duties, which had implications both for family mental health and staff well-being (45). As countries around the world begin vaccination programmes on an unprecedented scale, it is nevertheless likely that future pandemics will occur (46) and, in this context, lessons can be learned from those service areas that were able to more effectively support vulnerable families during the COVID-19 pandemic.

We also note that it took far longer for mental health services to implement remote digital technologies during the first lockdown when compared to the education and business sectors (this happened, in part, due to the redeployment of mental health clinicians to frontline COVID-19 duties). While there was commendable outreach by phone, the opportunities provided by digital technologies were not exploited in either NI or the RoI at the time. Encouragingly, considerable progress has been made in mental health remote provision during subsequent lockdowns, and research indicates that online mental health supports can be effective, particularly when blended with face-to-face contact, although not all clients may be able to access these due to unreliable broadband connectivity or a lack of confidence in using virtual platforms (47). A range of high quality online resources exist that could be more widely disseminated/promoted to clinicians and families with PMI, including COPMI and the Emerging Minds websites^3^, although more evidence on their effectiveness is required. Recommendations for optimal online delivery include: addressing issues of client engagement, identifying core components of evidence-based interventions when adapting to virtual delivery, and monitoring outcomes and effectiveness (47).

This study was limited by time/resource constraints which meant that we were unable to directly interview more family members, conduct full verbatim transcription, and administer a mental health questionnaire to enable comparison with population mental health during the first lockdown. Nevertheless, this study has important implications for policy and practise in eliciting important insights into how a key underserved population—families with parental mental illness—coped during COVID-19 restrictions, as well as highlighting the experiences of service providers in supporting these families. The findings also provide some useful pointers as to how services and governments might more effectively support vulnerable families in both the context of the current pandemic but also as we emerge from it, and into the future (see Table 1). Lastly, the experiences outlined here suggest that we have a unique opportunity to re-imagine how we structure our daily work and family lives to improve work-life balance in a post-pandemic world.

**Table 1:** Recommendations for mental health services in current pandemic and future non-pandemic contexts.

•Enable mental health services to become “pandemic ready”: that is, continue service provision during lockdowns and as we slowly emerge from them, use a blend of phone, online and in-person formats (using pandemic guidelines where appropriate)
•Develop/adapt/scale-up online interventions (e.g., psycho-educational videos) for use in both a pandemic and non-pandemic service context
•Incorporate more peer-based and community/voluntary sector provision of supports to provide for the medication needs of service users
•Minimise the redeployment of short-staffed mental health professionals to frontline pandemic duties. Instead, recruit dedicated testing/swabbing teams, as happened in Ireland 6 months into the COVID-19 pandemic
•Recognise the drawbacks of service overload and duplication
•Implement a “think family” approach to mental health service provision

## Data Availability Statement

The raw data supporting the conclusions of this article will be made available by the authors, without undue reservation.

## Ethics Statement

The studies involving human participants were reviewed and approved by Maynooth University Social Research Ethics Committee. The patients/participants provided their written informed consent to participate in this study.

## Author Contributions

SMcGi conceived of the original idea (and is also Principal Investigator of the PRIMERA research programme). MF and SMcGi led in drafting the manuscript. CM, AG, GD, MK, and MD contributed to manuscript revision and important intellectual content. CM led in collating, reviewing and synthesising the literature. MK, MD, MF, and CM co-ordinated service responses. SMcGa and MD liaised with service users.

## Acknowledgements

We would like to thank the clinicians and families involved in the PRIMERA research and from TFNI who contributed their feedback on their experiences during the COVID-19 lockdown.

## Conflict of Interest

The authors declare that the research was conducted in the absence of any commercial or financial relationships that could be construed as a potential conflict of interest.

## Publisher's Note

All claims expressed in this article are solely those of the authors and do not necessarily represent those of their affiliated organizations, or those of the publisher, the editors and the reviewers. Any product that may be evaluated in this article, or claim that may be made by its manufacturer, is not guaranteed or endorsed by the publisher.

## Footnotes

^1^COPMI - Children of Parents with Mental Illness.

^2^PRIMERA - **P**romoting **R**esearch and **I**nnovation in **M**ental h**E**alth se**R**vices for f**A**milies and children.

^3^ https://emergingminds.com.au/

## Abbreviations

AMHS: adult mental health services
CAMHS: child and adolescent mental health services
COPMI: children of parents with mental illness
NI: Northern Ireland
PMI: parental mental illness
PRIMERA: Promoting Research and Innovation in Mental hEalth seRvices for fAmilies and children
RoI: Republic of Ireland
TFNI: Think Family initiative in Northern Ireland.
