# Supplementary material for: Covid-19 and Families With Parental Mental Illness: Crisis and Opportunity
Source: Front Psychiatry. 2021 Jul 27;12:567447. doi: 10.3389/fpsyt.2021.567447 (PMC8353101; doi:10.3389/fpsyt.2021.567447)
Supplement: Supplementary file 1 [file Data_Sheet_1.docx]

**Supplementary table – sample quotes**

| **Themes & subthemes** | **Sample quotes** |
| --- | --- |
| **Improved family mental health or no change during lockdown** |  |
| Respite from stresses of daily life and time to connect with family | *“He has opened up so much about how he feels about us separating and that is definitely due to the time we’re spending together now”. (Parent)*  *“I didn’t realise how much stress commuting was doing to us until it stopped.” (Parent)*  *“Taking away the daily pressures of school, exams and work means that families are able to connect better with each other. There are less triggers for conflict, and when there is conflict, they seem more able to reach a positive resolution. For some parents they have become more confident in themselves, more empowered. This seems to be good for everyone’s mental health.”(Clinician, RoI)* |
| A chance to feel normal | *“I’m surprised by how well I’m doing and even better than some people I know who don’t attend [mental health] services. I can just be myself, take time to look after myself... And I feel less judged. The phone calls with my addiction counsellor are a good help.”* (Parent)  *“The recognition that the crisis is having a psychological impact on the general population has given some parents permission to discuss their own mental health needs more openly, with Covid as the protagonist.”* (Clinician, RoI) |
| No change | “I wouldn’t say things are better or worse. My husband still goes out to work. It’s hardest on my eldest boy who wants to meet his friends.” (Parent) |
| **Families struggling during lockdown** |  |
| Increased family distress due to worsening parental mental illness | *“Families with one parent are finding this period very difficult especially if they feel they’re relapsing. They have the added worry of who is going to look after the childre*n.” (Clinician, NI)  *“Where a parent has emotional dsyregulation, they are struggling with the restrictions and not being able to get respite from children and home life.”* (Clinician, RoI) |
| Escalating child misbehaviour | *“I didn’t think it was going to be this hard. With the schools closed, I can’t manage the [five] children. We are literally breaking apart...Tusla are involved.”* (Parent) |
| Protecting others from increased stress | *“We are finding it very hard financially with me not allowed to work and schools closed. The kids are not doing too bad but we [parents] are struggling.”* (Parent) |
| Coercive controlling behaviour | *“It’s awful. He goes out drinking with his mates and I am left here with the baby and the risk of infection. I have no way out.”* (Parent) |
| **Service responses and staff wellbeing** | *“We are providing 'essential' services which is extremely important for public health safety due to COVID-19, but does feel inadequate from a mental health perspective.”* (Clinician, RoI)  *“Foster placements are breaking down with no crisis intervention, and young people are being transferred to residential care. Social workers are overwhelmed.”* (Clinician, RoI).  *“Telephone support has been received well by most service users. We have been able to provide more intensive telephone support as the rate of new referrals to adult mental health services has more than halved since the Covid restrictions. We have also been able to arrange home visits and out-patient clinics where required, using Covid guidelines.”* (Clinician, RoI) |
|  |  |
